# Contribution of HIF-1 and drug penetrance to oxaliplatin resistance in hypoxic colorectal cancer cells

**DOI:** 10.1038/sj.bjc.6605311

**Published:** 2009-09-15

**Authors:** D L Roberts, K J Williams, R L Cowen, M Barathova, A J Eustace, S Brittain-Dissont, M J Tilby, D G Pearson, C J Ottley, I J Stratford, C Dive

**Affiliations:** 1 Clinical and Experimental Pharmacology Group, Paterson Institute for Cancer Research, University of Manchester, Wilmslow Road, Manchester M20 4BX, UK; 2 Experimental Oncology, School of Pharmacy and Pharmaceutical Sciences, Stopford Building, University of Manchester, Oxford Road, Manchester M13 9PT, UK; 3 Northern Institute for Cancer Research, Paul O'Gorman Building, Medical School, Framlington Place, University of Newcastle, Newcastle upon Tyne NE2 4HH, UK; 4 Science Labs, Department of Geological Sciences, Durham University, Durham DH1 3LE, UK

**Keywords:** hypoxia, apoptosis, DNA damage, oxaliplatin, tumour spheroids, bioavailability

## Abstract

**Background:**

Hypoxia is as an indicator of poor treatment outcome. Consistently, hypoxic HCT116 colorectal cancer cells are resistant to oxaliplatin, although the mechanistic basis is unclear. This study sought to investigate the relative contribution of HIF-1 (hypoxia-inducible factor-1)-mediated gene expression and drug penetrance to oxaliplatin resistance using three-dimensional spheroids.

**Methods:**

Hypoxia-inducible factor-1*α* function was suppressed by the stable expression of a dominant-negative form in HCT116 cells (DN). Cells were drug exposed as monolayer or multicellular spheroid cultures. Cells residing at differing oxygenation status were isolated from Hoechst 33342-treated spheroids using flow cytometry. Sub-populations were subjected to clonogenic survival assays and to Inductively-Coupled Plasma Mass Spectroscopy to determine oxaliplatin uptake.

**Results:**

In spheroids, a sensitivity gradient (hypoxic<aerobic) was revealed by survival assays and this correlated with levels of platinum-bound DNA. The resistance of hypoxic sub-populations exceeded relative changes in adduct levels, implicating factors other than drug penetrance in cell response. Dominant-negative monolayer cells showed no resistance to oxaliplatin in hypoxia and spheroids; the relative resistance of hypoxic compared with aerobic sub-populations was reduced compared with those from controls.

**Conclusion:**

Overall, data show that drug penetration, DNA damage levels and HIF-1-dependent processes, all contribute to the resistance of hypoxic cells to oxaliplatin.

Hypoxia, a common feature of human cancers, is often associated with poor prognosis (Brown and Wilson, 2004). Impaired drug penetration into hypoxic regions of tumours was thought to explain the poor prognosis, but recently an adaptive cellular response to hypoxia was demonstrated to reduce the cytotoxicity of many drugs. A central component of hypoxic adaptation is the stabilisation and activity of hypoxia-inducible factor-1 (HIF-1). Hypoxia-inducible factor-1 orchestrates the transcriptional regulation of genes involved in a plethora of cellular processes, including glycolysis, angiogenesis and metastasis. We previously demonstrated that activation of HIF-1 provoked changes in apoptosis pathway regulatory protein levels that affect drug sensitivity (Erler *et al*, 2004). Furthermore, inhibition of the HIF-1 function reduces hypoxia-mediated radio- and chemo-resistance (Moeller *et al*, 2004; Williams *et al*, 2005; Brown *et al*, 2006; Moeller and Dewhirst, 2006). Consequently, HIF-1 is perceived as a tractable drug target (Stratford *et al*, 1994; Williams *et al*, 2004; Semenza, 2007), and novel anticancer agents targeted to HIF-1-modulated pathways are being evaluated for clinical use.

Multicellular spheroids that model the hypoxic microenvironment *in vivo* can be exploited to achieve a more comprehensive assessment of factors contributing to resistance of hypoxic cells within tumours (Jones *et al*, 1982; West *et al*, 1984; Olive *et al*, 1996). Moreover, isolation of cell sub-populations from different spheroid microenvironments allows a detailed mechanistic study (Olive *et al*, 1996). Here, monolayer and spheroids of HCT116 colorectal cancer (CRC) cells treated with oxaliplatin were compared with dissect contributions of drug bioavailability (reported by DNA adducts), hypoxia and HIF-1 function(s) to the cellular response to a clinically relevant therapy for CRC.

## Materials and methods

All materials were from Sigma-Aldrich (Gillingham, UK) unless otherwise stated.

### Cell culture and drug treatment

HCT116 human CRC cells were cultured under standard conditions and were exposed to hypoxia for a minimum of 8 h before drug treatments (details in supplementary methods). Spheroids were formed as detailed in supplementary methods. Spheroids (∼50) were placed into 3.6 ml cryotubes (Nunc, Fisher Scientific, Loughborough, UK) with 1 ml media containing drug or vehicle. The tubes were gassed with 5% CO_2_/air, sealed and incubated at 37°C on a Stuart roller mixer device (VWR International, Lutterworth, UK). Oxaliplatin (Alexis Biochemicals via Axxora (UK) Ltd, Birmingham, UK) was prepared as a 10 mM stock solution in PBS, stored as aliquots at −80°C, and was only subjected to a single freeze-thaw cycle. To treat cells or spheroids, the stock solution was diluted in media to the appropriate concentration.

### Generation and validation of cell lines to investigate HIF-1

The coding sequence of a dominant-negative (DN) HIF-1*α* construct (Brown *et al*, 2006) was cloned into pEFIRESp (Hobbs *et al*, 1998). Growing phase HCT116 or HT1080 cells were transfected with DN or empty vector (EV) using lipofectamine according to the manufacturer's instructions (Invitrogen, Paisley, UK), with puromycin added 48 h later. Resistant colonies were expanded and HIF function was assessed by HRE reporter assay (Brown *et al*, 2006). Further validation was carried out by immunoblotting cell extracts obtained after aerobic or hypoxic conditions for HIF-1*α* and the HIF-1 downstream target glucose transporter-1 (GLUT-1) using the following primary antibodies: HIF-1*α* (1 : 500; BD Transduction Laboratories, Oxford, UK), GLUT-1 (1 : 6000, AB1340, Chemicon, Watford, UK) and *β*-actin (1 : 80 000; Sigma—Aldrich), followed by horseradish peroxidase-coupled secondary IgG with resolution by enhanced chemiluminescence.

Cells reporting HIF function were generated by transfecting HCT116 cells with an LDH-HRE-SV40 min-luciferase construct in pCI-neo and selecting stable colonies after G418 exposure. Colonies exhibiting robust HRE-mediated luciferase induction after hypoxic exposure were transfected with pEFIRESp encoding renilla luciferase, selected using puromycin and monitored for HRE-firefly luciferase and constitutive renilla luciferase expression using the dual-luciferase reporter assay following the manufacturer's guidelines (Promega, Southampton, UK).

### Immunohistochemistry

Spheroids were fixed in 4% buffered formalin overnight at 4°C and paraffin embedded. Sections were cut (∼4 *μ*m) and immunostaining was performed after dewaxing in xylene and rehydration in graded alcohols. Antigen retrieval was carried out by microwaving (800 W, 20 min) and cooling (15 min) in 10 mM citrate buffer, pH 6.0. Sections were blocked (0.1 M Tris-HCl, 0.15 M NaCl, pH 7.5, 4% normal goat serum (Dako, Glostrup, Denmark) for 2 h and incubated overnight at 4°C with primary antibody or antibodies: cleaved caspase-3, (1 in 100, CC3, Cell Signalling Technologies, Hitchin, UK), Ki67 (1 in 100, Clone MIB-1, Dako, Ely, UK) or GLUT-1 (1 in 100, Autogen Bioclear, Calne, UK). Negative controls consisted of substituting the primary antibody with mouse IgG1 or rabbit IgG at the same dilution. Sections were incubated with Alexa-conjugated donkey anti-mouse or donkey anti-rabbit immunoglobulin for 2 h (1 in 1000, Invitrogen), and then mounted using Prolong Gold Antifade containing DAPI (Invitrogen). Slides were viewed and images captured using an Olympus BX51 microscope. The intensity for each fluorescence channel was plotted from the necrotic core's edge to the outer rim using ‘plot profile’ in ImageJ (Wayne Rasband, NIH, Bethesda, MD, USA). Fluorescence intensity for CC3 and Ki67 staining was normalised to DAPI (accounting for changes in cellularity) and distances were converted into % penetrance.

### Cell viability and clonogenicity

Cells were plated either in T25 flasks (5 ml media, Corning, Amsterdam, The Netherlands) for clonogenic assays or in 96-well plates (100 *μ*l media per well, Falcon, via Scientific Laboratory Supplies, Nottingham, UK), and were allowed to adhere for 16 h before incubation in standard atmospheric (5% CO_2_/air) or hypoxic conditions for 8 h before drug treatment. Cells were maintained in hypoxic or standard atmospheric conditions and drugs administered for 16 h. Media were replaced and cells incubated under normoxia for 6 days before SRB assay as previously described (Vichai and Kirtikara, 2006), and the concentration that reduced growth by 50% (IC_50_) was determined. Clonogenicity after a 16 h drug exposure was determined as previously described (Williams *et al*, 2002). IC_50_ was the concentration resulting in a surviving fraction of 0.5 compared with untreated cells.

### Fluorescence-activated cell sorting

Spheroids were washed with fresh media and incubated (20 min, 37°C) with 10 *μ*M Hoechst 33342 (Invitrogen). After a PBS wash, spheroids were dissociated with trypsin (Invitrogen, 0.5%, 10 min), centrifuged and resuspended in fresh media at 10^7^ cells ml^−1^ before sorting into four fractions on the basis of Hoechst 33342 fluorescence using a BD FACSAria (Becton Dickinson, Oxford, UK). Debris was gated out and the brightest (B) and dimmest (D) 15% cells were sorted, with the remaining cells being equally distributed into medium-dim (MD) and medium-bright (MB) fractions. Sorted fractions were plated for clonogenic survival, assayed for luciferase activity (reporter cell lines) or used for determination of platinum (Pt) adduct levels after drug treatment. The oxaliplatin concentration required to kill 90% cells in each sorted sub-population was compared with that required to kill 90% of the bright (B), outermost fraction. A relative resistance value was calculated as the dose modification factor (DMF) for 90% cell kill (ratio of dose required to kill 90% cells in the inner-fractions compared with that in the B fraction).

### Oxaliplatin adduct quantification

Total intracellular Pt levels were determined from monolayers or from sorted spheroid sub-populations. Cells were resuspended in ice-cold 10 mM Tris-HCl, 1% Triton-X100 pH 7.5 containing protease inhibitor cocktail and were sonicated to lyse cells and fragment DNA. Proteins were hydrolysed with NaOH and the remaining DNA was hydrolysed with nitric acid. Samples were stored at −80°C. Nitric acid (67–70%) was Optima grade for trace metal analysis (Fisher Scientific). Cellular Pt content was determined using a magnetic sector ICP-MS (Thermo Element 2) with a low uptake nebuliser. To correct for and measure any signal suppression due to sample matrix effects, all standards, blanks and samples contained thallium (Tl) as internal standard; hafnium (Hf) was also monitored as described previously (John *et al*, 2003). Platinium concentrations were derived by reference to a standard solution calibration curve using the mean concentration from the three Pt isotopes.

### Statistical analysis

Two-tailed *t*-tests were used to evaluate significant differences. *P*-values of <0.05 were considered as statistically significant.

## Results

**Figure 1:**
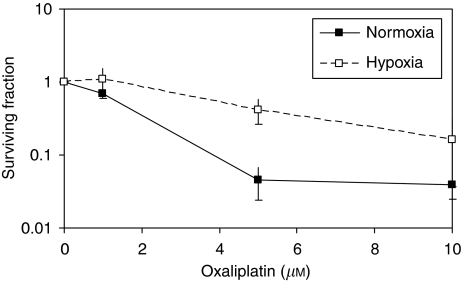
Hypoxia-induced oxaliplatin resistance. HCT116 cells were exposed to normoxia or hypoxia for 8 h and were treated with oxaliplatin (0–25 *μ*M) for 16 h under continued normoxia or hypoxia. Clonogenic survival was assessed 6–10 days later. The surviving fraction was determined from the mean of two experiments, each containing a range of cell densities in duplicate.

Previous studies established that CRC cells resist a number of chemotherapeutics under hypoxic conditions (Erler *et al*, 2004). In this study, hypoxic HCT116 cells were found to be resistant to oxaliplatin using short-term SRB (IC_50_ normoxia=5.2 *μ*M *vs* IC_50_ hypoxia=10.1 *μ*M, *P*=0.015) and clonogenic survival end points (IC_50_ normoxia 2.4 *μ*M *vs* IC_50_ hypoxia 5.9 *μ*M; Figure 1). The IC_50_ for oxaliplatin under hypoxia was 1.9- and 2.4-fold higher than that in normoxia for SRB and clonogenic assays, respectively.

### Hypoxic cells within multicellular CRC spheroids were oxaliplatin resistant

**Figure 2:**
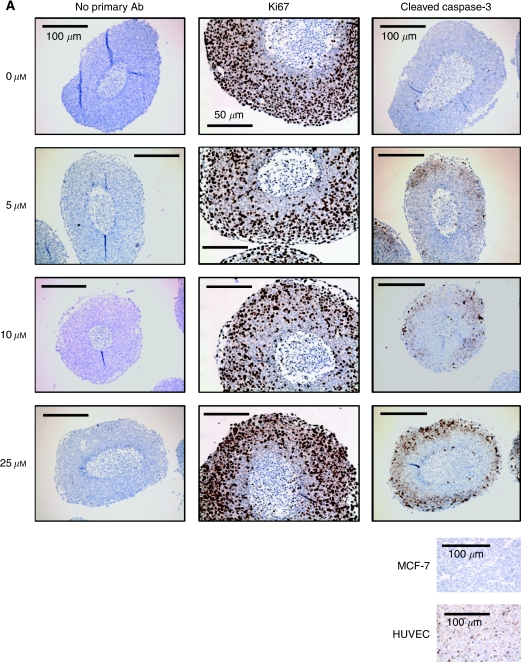

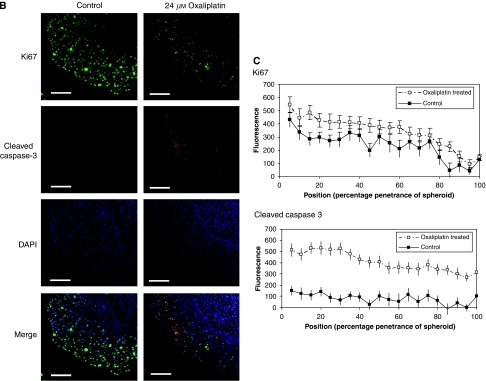
The effect of oxaliplatin on proliferation and apoptosis in spheroids. Spheroids were treated with oxaliplatin (0–25 *μ*M) for 24 h before fixation, processing and immunohistochemical assessment of Ki67 or cleaved caspase-3 (CC3) (**A**). Specificity of CC3 staining was confirmed using positive and negative cell pellets. The staining shown is representative of three separate experiments. Scale bars indicate either 100 *μ*m (controls and CC3) or 50 *μ*m (Ki67). Studies were repeated using immunofluorescence (**B**), and localisation of biomarkers was confirmed by the assessment of fluorescent intensity across spheroid sections (**C**). Sections were stained for Ki67 (green) and CC3 (red) with DAPI (blue) counterstaining to identify nuclei (**B**). Scale bars=50 *μ*m. The fluorescent profile in each channel was plotted from the necrotic core to the outer rim and compared between treated and control sections to correlate staining with positional information (**C**). Plots are the mean intensity of 14 spheroids for each condition.

To investigate cellular response to chemotherapy in a more physiologically relevant model, HCT116 cells were grown as three-dimensional spheroids. Figures 2A and B show that untreated spheroids have a high proportion (>90%) of proliferating cells (Ki67 positive), except in peri-necrotic regions (<15% positive), whereas apoptosis (CC3 positivity) was observed in <10% cells (*n*=10). Treatment with 5–10 *μ*M oxaliplatin decreased Ki67 staining. At 25 *μ*M, oxaliplatin increased Ki67 staining, a paradoxical result most likely explained by nuclear fragmentation. An oxaliplatin concentration-dependent increase in CC3 staining was observed to be restricted to the outer rim, suggesting that response of inner cells may be dependent on their oxygenation and/or nutrient status and/or drug penetration through the cell mass. Similar results were observed using dual staining; Ki67 stained in outer and peri-necrotic regions, whereas CC3 was only evident in outer regions (Figure 2B). Staining specificity was ascertained using MCF-7 cell pellets (caspase-3 null) and staurosporine-treated HUVEC cells as negative and positive controls, respectively.

**Figure 3:**
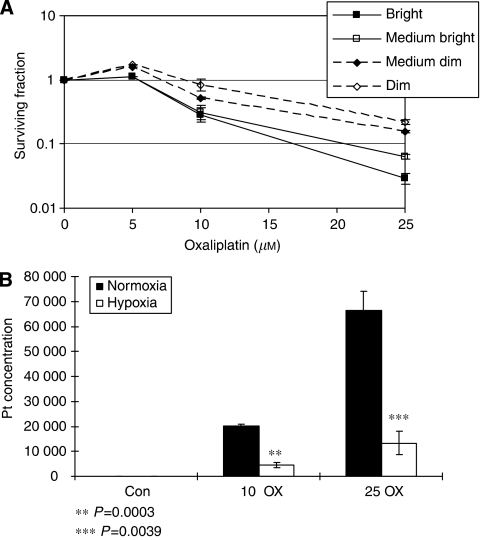
Effect of oxaliplatin on cell survival in spheroids and quantification of platinum adducts. Spheroids were treated with oxaliplatin (0–25 *μ*M) for 1 h and then fractionated. Clonogenicity was determined and the surviving fraction plotted for each sub-population and oxaliplatin concentration (**A**). Monolayer samples were assessed for total platinum levels by ICP-MS (**B**). Platinum values are given as fmoles mg^−1^ protein. Data are the mean of three experiments (±s.e.).

To investigate the relationship between proliferation, cell death and cellular location, staining was repeated on oxaliplatin-treated spheroids using immunofluorescent detection for direct comparison of respective biomarkers (Figure 2B and C). Ki67 staining decreased significantly towards the necrotic core (the outermost 10% of the spheroid compared with the innermost 10%, *P*<0.0001), and this pattern did not change with oxaliplatin treatment. Staining for CC3 was significantly higher in the outer 50% cells within treated spheroids (*P*<0.0001) compared with the inner 50%. Cell sub-populations were isolated to assess any relationship between histology and cell survival. After exposure to oxaliplatin for 1 h, spheroids were treated with Hoechst 33342, disaggregated and sorted into four sub-populations: bright (B), medium-bright (MB), medium-dim (MD) and dim (D). Those cells with high fluorescence (B) come from the outer, well-oxygenated regions, whereas cells showing low fluorescence (D) arise from inner, hypoxic regions (Olive *et al*, 1985). Consistent with the immunohistochemistry analysis, Figure 3A shows that D and MD sub-populations had significantly increased IC_50_ values compared with B sub-population cells (values were calculated from raw data for each experiment and averaged to give IC_50_ (D), 19.67 *μ*M, (MD) 17.45 *μ*M compared with (B) 13.0 *μ*M, *P*=<0.0001). Increasing oxaliplatin exposure from 1 to 24 h revealed a similar trend (data not shown). These data also demonstrate that culture of cells in a three-dimensional system alters their response to oxaliplatin, as revealed by comparing the outer cell response to that of control monolayers; this reveals that cells grown in three dimensions are inherently more resistant (B sub-population IC_50_, 13 *μ*M, control monolayer IC_50_, 2.4 *μ*M).

### Reduced drug uptake/retention in hypoxic cells within spheroids did not account fully for reduced cytotoxicity

To investigate whether the change in cell survival throughout spheroids was due to differential oxaliplatin bioavailability, Pt-adduct formation was analysed in sorted sub-populations using Inductively Coupled Plasma Mass Spectroscopy (ICP-MS; Table 1). After exposure to oxaliplatin (25 *μ*M, 2 h), cells were sorted into D, MD, MB and B sub-populations and adducts were expressed as % Pt-adducts in the B sub-population. Adduct formation was reduced by 1.3-fold and 1.5-fold in MD and D cells, respectively, compared with that observed in the B sub-population. However, survival was increased after exposure to oxaliplatin from SF 0.02 for the B sub-population to 0.157 for MD and 0.22 in D sub-populations (increasing 7.8-fold from B to MD and 11-fold from B to D, Figure 3A).

To investigate whether the observed changes in Pt-adduct formation merely reflected oxaliplatin penetrance through spheroids, HCT116 monolayers were treated with oxaliplatin, and adduct formation was analysed after 21 or 1% oxygen exposure. Interestingly, adduct formation was also reduced significantly in hypoxia (*P*=0.013 at 10 *μ*M, *P*=0.026 at 25 *μ*M) in which there was no biophysical hindrance to drug exposure (Figure 3B). Taken together with relative survival data (Figure 3A), this suggests that mechanisms other than drug penetrance contribute to oxaliplatin resistance in hypoxic cells within spheroids.

### Monolayer cultures of HCT116 that lack HIF function did not show hypoxic resistance to oxaliplatin

**Figure 4:**
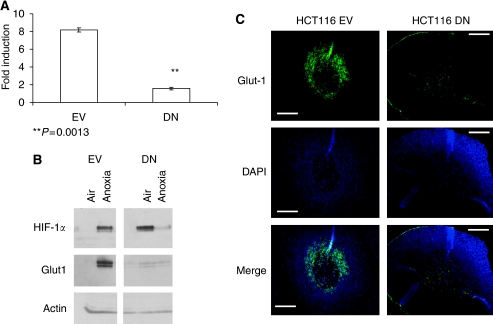
Validation of the HCT116 DN model. HCT116 cells were stably transfected with an empty vector cassette (EV) or an hypoxia-inducible factor (HIF)-*α* dominant-negative construct (DN). Altered HIF-1 function was validated using an HIF reporter assay analysed in the presence or absence of hypoxia mimetic cobalt chloride (**A**) and by immunoblot analysis of the HIF-1 target glucose transporter-1 (GLUT-1) (**B**). At ∼500 *μ*m, HCT116 wild type, EV or DN spheroids were treated with 100 *μ*M pimonidazole, fixed, sectioned and immunostained for GLUT-1 and pimonidazole adducts (**C**). Data are from a representative experiment showing the mean ±s.d. of triplicate samples (**A**); immunoblots (**B**) and immunostaining (**C**) are representative of three separate experiments. Scale bars indicate 100 *μ*m.

**Figure 5:**
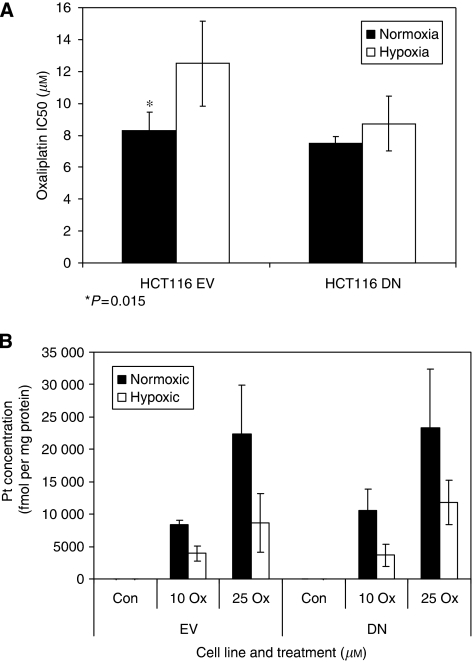
Hypoxic resistance was hypoxia-inducible factor (HIF) dependent and unrelated to platinated-DNA adduct formation. Empty vector (EV) and dominant-negative (DN) cells were exposed to oxaliplatin under standard atmospheric or hypoxic conditions, and IC_50_ values were calculated from SRB assays (**A**). Total cellular platinum concentrations were determined in control or hypoxic EV and DN cells after oxaliplatin exposure (**B**). Data are the mean of three experiments ±s.e.

Our previous studies using monolayers implicated HIF-1 in hypoxic resistance to chemotherapeutics (Erler *et al*, 2004; Brown *et al*, 2006). To facilitate the extrapolation of these observations to spheroids, stable HCT116 cell lines were generated to express a DN variant of HIF-1*α* (Brown *et al*, 2006). Control cells expressed an EV. Expression of DN HIF-1*α* ablated HIF-1-mediated reporter gene expression after exposure to cobalt chloride (Figure 4A), as well as the expression of the HIF-1 target GLUT-1 (Figure 4B). Expression of native HIF-1*α* protein was unaffected by the DN variant, but hypoxia-induced expression of GLUT-1 was absent from DN cells grown as monolayers or as spheroids (Figure 4B and C). In EV spheroids, GLUT-1 localised to the hypoxic, peri-necrotic areas corresponding to regions labelled by the hypoxia biomarker pimonidazole (Figure 4C, data not shown). Glucose transporter-1 staining was not seen in hypoxic regions of DN spheroids (Figure 4C). Exposure of monolayer EV cells to oxaliplatin revealed hypoxic resistance by SRB assay (Figure 5A), which matched that of parental cells (IC_50_ hypoxia/IC_50_ air 1.4-fold, not shown). However, no hypoxic resistance to oxaliplatin was observed in DN cells, demonstrating an HIF-1-dependent resistance mechanism (Figure 5A). Similar results were observed on inhibition of HIF-1 function through DN expression in HT1080 human fibrosarcoma cells exposed to 5 *μ*M oxaliplatin for 24 h (Supplementary Figure 1).

### The differences in response between HCT116 cells with and without functional HIF was independent of oxaliplatin uptake

To ensure that changes in oxaliplatin sensitivity were not due to differential drug uptake between EV and DN cells, Pt-adduct formation was assessed in monolayers exposed to a range of oxaliplatin concentrations under normoxia or hypoxia (Figure 5B). A concentration-dependent increase in protein-Pt-adduct formation was revealed in EV and DN cells under both conditions. The adduct formation was reduced in hypoxia *vs* normoxia for both cell lines, consistent with data from untransfected cells (Figure 3), suggesting that modification of oxaliplatin uptake/retention under hypoxic conditions is independent of HIF function.

### Lack of HIF function ablated hypoxic resistance to oxaliplatin in cells derived from spheroids

**Figure 6:**
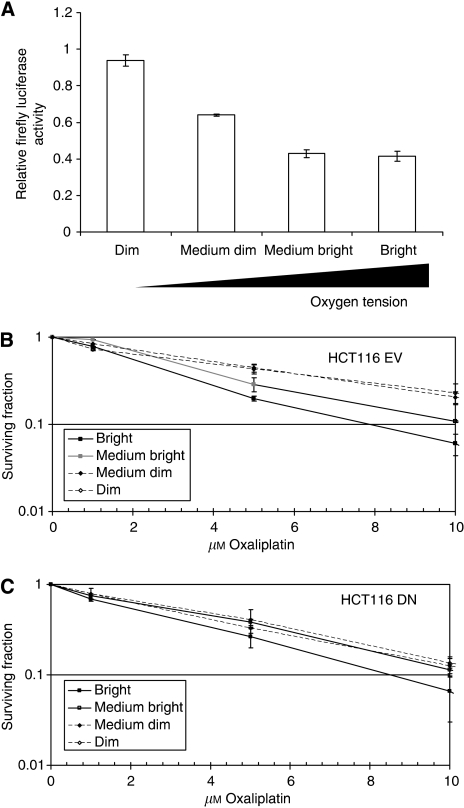
Hypoxia-inducible factor (HIF) activity correlated with oxaliplatin resistance in spheroid cultures. A HCT116 reporter cell line stably expressing a constitutive renilla luciferase and hypoxia-induced (LDH-HRE) firefly luciferase was used to validate the fractionation methodology. Hypoxia-inducible factor-1 activity was measured in sorted spheroid sub-populations by determining the relative expression of firefly to renilla luciferase (**A**). HCT116 EV (**B**) and DN (**C**) spheroids were fractionated on the basis of Hoechst 33342 fluorescence and the resulting sub-populations were exposed to various concentrations of oxaliplatin. Cell survival was assessed by clonogenic assay. Data are the mean of three experiments ±s.e.

Oxaliplatin sensitivity was investigated in sorted sub-populations of EV and DN spheroids. Initial studies were undertaken to validate whether sub-populations showed differences in HIF activity using spheroids of HCT116 HIF-1 reporter cells. These cells express firefly luciferase under the transcriptional regulation of HIF-1 (through inclusion of a trimer sequence of the lactase dehydrogenase promoter) and a renilla luciferase construct under the regulation of the strong constitutive promoter elongation factor-1*α*. The expression level of firefly luciferase relative to renilla luciferase in each sub-population was compared with that obtained from whole-spheroid lysates (Figure 6A). The relative level of HIF-1-driven firefly luciferase expression compared with that of renilla luciferase was 56% lower in the outer spheroid regions compared with the inner region (D), and increased in a step-wise manner to the inner regions (Figure 6A), confirming that spheroid sub-populations differed in their HIF-transactivation levels.

Empty vector and DN spheroids were exposed to oxaliplatin after sorting to exclude potential changes in penetrance that could confound HIF-mediated effects. The response of the outer two fractions (B and MB) was almost identical for both cell types (Figure 6B and C), with greater sensitivity observed in outermost cells. However, whereas the inner two sub-populations (D and MD) of EV spheroids showed resistance to oxaliplatin treatment, this was not apparent in DN cells isolated from the same sub-populations. Relative resistance factors were calculated for the sub-populations by determining the DMF required for 90% kill compared with that of the B sub-population. The DMFs for MB, MD and D populations were 1.4, 1.9 and 2.1, and 1.3, 1.4 and 1.4 for the EV- and DN-derived spheroid populations, respectively (Figure 6B and C, data not shown). As during this experiment cells were re-oxygenated during the period of drug exposure, we investigated how re-oxygenation affected the hypoxia-induced changes in the levels of GLUT-1 (upregulated by HIF-1) and Bax (repressed by hypoxia independent of HIF-1) at the protein level (Supplementary Figure 2). This revealed that changes in protein levels were maintained for 8–12 h on re-oxygenation, indicating that our sorted fractions maintained a hypoxic ‘phenotype’ during post-sort drug exposures.

## Discussion

This study investigated oxaliplatin sensitivity in HCT116 CRC cells using both monolayers and spheroids to identify relative contributions of physical *vs* biological mechanisms of hypoxic resistance to chemotherapy. In monolayers, in which issues of exposure and biodistribution are eliminated, oxaliplatin was less effective in hypoxic compared with aerobic cells. Interestingly, Pt-adduct formation was reduced markedly in hypoxic cells exposed to oxaliplatin, which could contribute to the observed differential toxicity. Treatment of cells with oxaliplatin at concentrations greater than clonogenic IC_50_ suggested that apoptosis was dependent on the generation of reactive oxygen species (Berndtsson *et al*, 2007), which would be compromised in hypoxia.

Consistent with data from monolayers, sensitivity to oxaliplatin treatment was greater in cells of the outer, better-oxygenated regions of HCT116 spheroids. Oxaliplatin provoked concentration-dependent increases in apoptosis in outer regions, whereas it had minimal impact on proliferation throughout the spheroid. When spheroids were fractionated after oxaliplatin treatment, clonogenic survival observed in outer region cells was eightfold higher than that in innermost cells. The difference in Pt-adduct formation between these two sub-populations was lower (1.5-fold reduction). This suggests that compromised bioavailability was not the major mechanism underpinning the poorer response of hypoxic cells to oxaliplatin treatment. Furthermore, it suggests that in more physiologically relevant culture models, cellular ‘handling’ of oxaliplatin is markedly different to that observed in monolayers and that in these models, adduct formation is not a reliable biomarker for cytotoxicity *per se*.

In this study, a genetic approach was used to inhibit HIF-1 function in HCT116 cells by the introduction of a DN HIF-1*α* subunit. Oxaliplatin was equitoxic to control monolayers of DN or EV cells. However, whereas hypoxia induced resistance in EV cells, in DN cells hypoxic sensitivity was equivalent to that in air. Oxaliplatin adduct formation was unaltered by ablated HIF function. In fractionated spheroids, the outermost cells exhibited similar responses to oxaliplatin (SF 10 *μ*M oxaliplatin 0.061 EV *vs* 0.066 for DN), consistent with monolayers. However, cells from the inner regions of DN spheroids were more sensitive than were their EV counterparts. The level of sensitisation within spheroid sub-populations correlated with HIF-1 activity determined using fractionated sub-populations from HCT116 cells expressing an HIF-1 responsive-luciferase reporter.

Taken together, these data suggest that HIF-1 has an important role in mediating oxaliplatin sensitivity in HCT116 cells. Given our previous findings, as well as those of others, hypoxic cell chemo-resistance is predicted to be a strong contributor to the poor response of CRC to standard therapies. A potential contribution of HIF-1 to this phenotype is supported by clinical studies demonstrating HIF-1*α* expression as a poor prognostic indicator in CRC (Lu *et al*, 2006). The mechanisms by which HIF-1 contributes to resistance are likely to be complex and varied depending on cell genotype. Indeed, recent data observing sensitisation to cisplatin using HIF-targeting RNAi suggest that reversal of hypoxic resistance is p53 dependent (Hao *et al*, 2008). We previously reported that HIF-1 can alter the balance of pro- and anti-apoptotic proteins under hypoxic conditions (Erler *et al*, 2004). Specifically, HIF-1 can downregulate pro-apoptotic Bid, the reversal of which is associated with enhanced chemotherapy response (Brown *et al*, 2006). The reciprocal relationship between HIF-1*α* and Bid expression is maintained in clinical samples of CRC, supporting its potential importance (Seenath *et al*, 2008). With current interest in the development of HIF-targeting agents as novel cancer therapies, these data would support their exploitation in combination with standard chemotherapy to enhance the treatment response in CRC.

## Figures and Tables

**Table 1:** Distribution of platinum (Pt)-adduct formation in HCT116 spheroids

	**Bright**	**Medium-bright**	**Medium-dim**	**Dim**
Percentage (%) Pt	100	95.994	76.361	66.020
s.e.	—	4.615	8.839	4.333
*P*-value *vs* bright	—	0.4344	0.0555	0.0014
