# Supplementary material for: Contribution of HIF-1 and drug penetrance to oxaliplatin resistance in hypoxic colorectal cancer cells
Source: Br J Cancer. 2009 Sep 15;101(8):1290–7. doi: 10.1038/sj.bjc.6605311 (PMC2768443; doi:10.1038/sj.bjc.6605311)
Supplement: Supplementary Methods [file 6605311x1.doc]

**Supplementary Figures.** Roberts, DL *et al*

**Figure S1. Hypoxia mediated Oxaliplatin resistance is reduced by HIF-1 inhibition in HT1080 cells.** HT1080 cells were cultured for 8 h under normoxic or anoxic (<0.01% O_2_) conditions prior to 24 h of treatment with a 5 μM of oxaliplatin (with continued normoxic or anoxic incubation). Cell survival was then assessed by clonogenic assay and colonies were counted after 10 days. Values are means ± S.E.M. of 3 experiments (DN1 p = ≤ 0.05 and DN2 p = ≤ 0.01 versus WT cells).

**A**

**B**

Re-oxygenation

6h

Normoxia

Anoxia

Re-oxygenation

12h

**Figure S2. Hypoxia mediated protein changes are maintained following re-oxygenation.**

HCT116 cells were grown in 6 well plates and exposed to either 16h hypoxia (1% O_2_), anoxia (<0.001% O_2_) or standard atmospheric conditions (21% O_2_) prior to the start of the experiment. Cells were then moved from hypoxia and returned to standard atmospheric conditions for the times indicated. (A) Cells were then analysed by western blot for levels of HIF-1α, Bax and actin. This revealed that Bax levels remain repressed for over 8 h following re-oxygenation of cells whereas HIF-1α returned to normoxic levels in under 2 h. (B) Cells were formed into pellets and processed for IHC analysis. Sections were then stained for GLUT-1 (brown colouration, magnification x400).

**Supplementary Material and Methods**

**Cell culture and drug treatment**

HCT116 human CRC cells and HT1080 fibrosarcoma cells (European Collection of Cell Cultures) were grown in 20ml of RPMI 1640 media (Invitrogen, Paisley, UK) with 10% FBS (Biosera, East Sussex, UK), and 2mM Glutamine (HT1080 cells only) in T75 flasks (Corning, The Netherlands) at 37°C in water saturated 5% CO_2_/air. To achieve hypoxia, cells were incubated in an InVivo400 chamber (Ruskinn, Leeds, UK) at 1%O_2_/5%CO_2_/94%N_2_ at 37°C for a minimum of 8h prior to any treatments. To generate an anoxic environment 5% CO_2_/5% H_2_/90% N_2_ was passed over a palladium catalyst to remove residual oxygen resulting in an oxygen concentration of <0.01%. This was then used to gas a Bactron anaerobic chamber (Sheldon Manufacturing, Cornelius, OR) containing the cells.

Spheroids were formed by plating cells in 10 ml of media at 2x10^5^ cells/ml on 10cm^2^ dishes coated with 0.5% agarose. Spheroids formed over 72h to 70-100µm diameter were cultured further in 300ml of media in 500ml spinner flasks. The spinner flasks were gassed with 5% CO_2_/air and cultured at approximately 25rpm with replacement of 75% of the media and regassing of the flask every three days until the spheroids had achieved a diameter of ~500µm. Under such conditions, pO_2_ of cells varied from 10% O_2_ at the spheroid surface to 0.1% in the spheroid center (Acker et al., 1987; Vaupel, 1977).

**Immunoblotting**

Immunoblots to detect Bax, actin and HIF-1α were performed as previously described (Erler et al., 2004).

**Supplementary References**

Acker, H., Carlsson, J., Mueller-Klieser, W. & Sutherland, R.M. (1987). Comparative pO2 measurements in cell spheroids cultured with different techniques. *Br J Cancer*, **56,** 325-7.

Vaupel, P. (1977). Hypoxia in neoplastic tissue. *Microvasc Res*, **13,** 399-408.
